# 5-(4-Fluoro­benzyl­idene)-2,2-dimethyl-1,3-dioxane-4,6-dione

**DOI:** 10.1107/S1600536810033155

**Published:** 2010-08-21

**Authors:** Wu-Lan Zeng

**Affiliations:** a MicroScale Science Institute, Department of Chemistry and Chemical Engineering, Weifang University, Weifang 261061, People’s Republic of China

## Abstract

The title compound, C_13_H_11_FO_4_, was prepared by the reaction of 2,2-dimethyl-1,3-dioxane-4,6-dione and 4-fluoro­benz­alde­hyde in ethanol. The 1,3-dioxane ring adopts an envelope conformation. The crystal structure is stabilized by weak inter­molecular C—H⋯O hydrogen bonds.

## Related literature

For background information on the use of Meldrum’s acid (2,2-dimethyl-1,3-dioxane-4,6-dione) in organic synthesis, see: Kuhn *et al.* (2003 ▶); Casadesus *et al.* (2006 ▶). For a related structure, see: Zeng & Jian (2009 ▶).
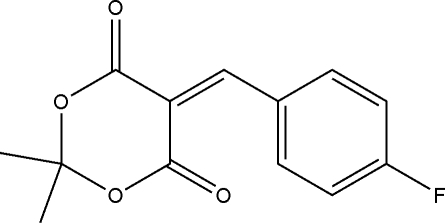


## Experimental

### 

#### Crystal data

C_13_H_11_FO_4_
*M* *_r_* = 250.22
Monoclinic, 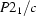

*a* = 10.607 (2) Å
*b* = 10.413 (2) Å
*c* = 11.366 (2) Å
β = 106.09 (3)°
*V* = 1206.2 (4) Å^3^
*Z* = 4
Mo *K*α radiation
μ = 0.11 mm^−1^
*T* = 293 K
0.17 × 0.15 × 0.10 mm

#### Data collection

Bruker SMART CCD diffractometer
11341 measured reflections
2748 independent reflections
1773 reflections with *I* > 2σ(*I*)
*R*_int_ = 0.042

#### Refinement

*R*[*F* ^2^ > 2σ(*F* ^2^)] = 0.048
*wR*(*F* ^2^) = 0.182
*S* = 1.16
2748 reflections
163 parameters
H-atom parameters constrained
Δρ_max_ = 0.16 e Å^−3^
Δρ_min_ = −0.24 e Å^−3^

### 

Data collection: *SMART* (Bruker, 1997 ▶); cell refinement: *SAINT* (Bruker, 1997 ▶); data reduction: *SAINT*; program(s) used to solve structure: *SHELXS97* (Sheldrick, 2008 ▶); program(s) used to refine structure: *SHELXL97* (Sheldrick, 2008 ▶); molecular graphics: *SHELXTL* (Sheldrick, 2008 ▶); software used to prepare material for publication: *SHELXTL*.

## Supplementary Material

Crystal structure: contains datablocks global, I. DOI: 10.1107/S1600536810033155/lh5116sup1.cif

Structure factors: contains datablocks I. DOI: 10.1107/S1600536810033155/lh5116Isup2.hkl

Additional supplementary materials:  crystallographic information; 3D view; checkCIF report

## Figures and Tables

**Table 1:** Hydrogen-bond geometry (Å, °)

*D*—H⋯*A*	*D*—H	H⋯*A*	*D*⋯*A*	*D*—H⋯*A*
C10—H10*A*⋯O1^i^	0.93	2.47	3.373 (3)	164

Symmetry code: (i) 
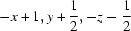
.

**Fig. 1.**
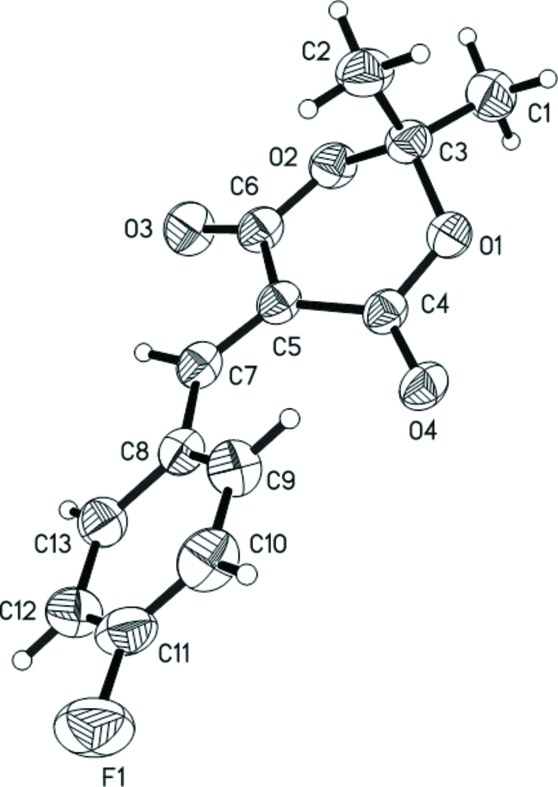
The molecular structure of (I), drawn with 30% probability ellipsoids and spheres of arbritrary size for the H atoms.

## supplementary crystallographic information

### Comment

Starting with its discovery and correct structural assignment, Meldrum's acid has become a widely used reagent in organic synthesis (Kuhn *et al.*, 2003; Casadesus *et al.*, 2006). We have recently reported the crystal structure of 5-(2-fluorobenzylidene)-2,2-dimethyl-1,3-dioxane-4,6-dione (Zeng & Jian, 2009). As part of our search for new Meldrum's acid derivatives the title compound,(I)(Fig. 1), has been synthesized and its crystal structure is reported herein. The crystal structure analysis confirms the title compound with atom C7 connected to a benzene ring via the C7-C8 single bond [1.451 (2)Å] and a 1,3-dioxane ring via the C7═C5 double bond [1.349 (2)Å]. The crystal structure is stabilized by weak intermolecular C—H···O hydrogen bonds (Table 1).

### Experimental

A mixture of malonic acid (6.24 g, 0.06 mol) and acetic anhydride(9 ml) in strong sulfuric acid (0.25 ml) was stirred with water at 303K. After dissolving, propan-2-one (3.48 g, 0.06 mol) was added dropwise into solution for 1 h. The reaction was allowed to proceed for 2 h. The mixture was cooled and filtered, and then an ethanol solution of 4-fluorobenzaldehyde (7.67g,0.06 mol) was added. The solution was then filtered and concentrated. Single crystals were obtained by evaporation of an petroleum ether-ethylacetate (3:1 *v*/*v*) solution of (I) at room temperature over a period of several days.

### Refinement

The H atoms were placed in calculated positions (C—H = 0.93–0.96 Å), and refined as riding with *U*_iso_(H) = 1.2*U*_eq_(C) or 1.5U_eq_(methyl C).

### Crystal data

**Table:**

C_13_H_11_FO_4_	*F*(000) = 520
*M**_r_* = 250.22	*D*_x_ = 1.378 Mg m^−^^3^
Monoclinic, *P*2_1_/*c*	Mo *K*α radiation, λ = 0.71073 Å
Hall symbol: -P 2ybc	Cell parameters from 2748 reflections
*a* = 10.607 (2) Å	θ = 3.0–27.5°
*b* = 10.413 (2) Å	µ = 0.11 mm^−^^1^
*c* = 11.366 (2) Å	*T* = 293 K
β = 106.09 (3)°	Block, colorless
*V* = 1206.2 (4) Å^3^	0.17 × 0.15 × 0.10 mm
*Z* = 4	

### Data collection

**Table:**

Bruker SMART CCD diffractometer	1773 reflections with *I* > 2σ(*I*)
Radiation source: fine-focus sealed tube	*R*_int_ = 0.042
graphite	θ_max_ = 27.5°, θ_min_ = 3.0°
φ and ω scans	*h* = −13→13
11341 measured reflections	*k* = −13→13
2748 independent reflections	*l* = −14→13

### Refinement

**Table:**

Refinement on *F*^2^	Primary atom site location: structure-invariant direct methods
Least-squares matrix: full	Secondary atom site location: difference Fourier map
*R*[*F*^2^ > 2σ(*F*^2^)] = 0.048	Hydrogen site location: inferred from neighbouring sites
*wR*(*F*^2^) = 0.182	H-atom parameters constrained
*S* = 1.16	*w* = 1/[σ^2^(*F*_o_^2^) + (0.1*P*)^2^] where *P* = (*F*_o_^2^ + 2*F*_c_^2^)/3
2748 reflections	(Δ/σ)_max_ < 0.001
163 parameters	Δρ_max_ = 0.16 e Å^−^^3^
0 restraints	Δρ_min_ = −0.24 e Å^−^^3^

### Special details

**Table:**

Geometry. All e.s.d.'s (except the e.s.d. in the dihedral angle between two l.s. planes) are estimated using the full covariance matrix. The cell e.s.d.'s are taken into account individually in the estimation of e.s.d.'s in distances, angles and torsion angles; correlations between e.s.d.'s in cell parameters are only used when they are defined by crystal symmetry. An approximate (isotropic) treatment of cell e.s.d.'s is used for estimating e.s.d.'s involving l.s. planes.
Refinement. Refinement of *F*^2^ against ALL reflections. The weighted *R*-factor *wR* and goodness of fit *S* are based on *F*^2^, conventional *R*-factors *R* are based on *F*, with *F* set to zero for negative *F*^2^. The threshold expression of *F*^2^ > σ(*F*^2^) is used only for calculating *R*-factors(gt) *etc*. and is not relevant to the choice of reflections for refinement. *R*-factors based on *F*^2^ are statistically about twice as large as those based on *F*, and *R*- factors based on ALL data will be even larger.

### Fractional atomic coordinates and isotropic or equivalent isotropic displacement parameters (Å^2^)

**Table:**

	*x*	*y*	*z*	*U*_iso_*/*U*_eq_	
O2	0.19531 (11)	0.04581 (14)	0.03108 (11)	0.0683 (4)	
O4	0.48662 (11)	0.09217 (13)	−0.12156 (11)	0.0669 (4)	
O1	0.29516 (11)	0.01292 (14)	−0.12672 (11)	0.0685 (4)	
C7	0.46120 (16)	0.27281 (18)	0.07462 (14)	0.0588 (4)	
H7A	0.4452	0.3029	0.1462	0.071*	
O3	0.27783 (14)	0.17669 (16)	0.18433 (12)	0.0848 (5)	
C5	0.38086 (15)	0.17577 (16)	0.02221 (13)	0.0541 (4)	
C4	0.39340 (15)	0.09646 (17)	−0.08040 (15)	0.0566 (4)	
C8	0.56723 (16)	0.34028 (17)	0.04331 (14)	0.0577 (4)	
C6	0.28220 (17)	0.13664 (19)	0.08633 (16)	0.0625 (5)	
C3	0.17201 (16)	0.0255 (2)	−0.09776 (16)	0.0661 (5)	
C13	0.65632 (18)	0.4046 (2)	0.13807 (17)	0.0693 (5)	
H13A	0.6460	0.4008	0.2166	0.083*	
C9	0.5822 (2)	0.3527 (2)	−0.07392 (17)	0.0706 (5)	
H9A	0.5226	0.3128	−0.1394	0.085*	
F1	0.86826 (14)	0.55357 (17)	−0.01890 (16)	0.1227 (6)	
C10	0.6833 (2)	0.4228 (2)	−0.0945 (2)	0.0837 (6)	
H10A	0.6928	0.4306	−0.1731	0.100*	
C2	0.0951 (2)	0.1370 (2)	−0.16850 (19)	0.0837 (6)	
H2A	0.1444	0.2149	−0.1462	0.126*	
H2B	0.0128	0.1447	−0.1494	0.126*	
H2C	0.0794	0.1222	−0.2547	0.126*	
C12	0.75946 (19)	0.4739 (2)	0.1185 (2)	0.0806 (6)	
H12A	0.8200	0.5144	0.1828	0.097*	
C11	0.7695 (2)	0.4809 (2)	0.0023 (2)	0.0814 (6)	
C1	0.1052 (2)	−0.1014 (2)	−0.1254 (2)	0.0918 (7)	
H1A	0.1597	−0.1669	−0.0775	0.138*	
H1B	0.0900	−0.1205	−0.2109	0.138*	
H1C	0.0229	−0.0986	−0.1058	0.138*	

### Atomic displacement parameters (Å^2^)

**Table:**

	*U*^11^	*U*^22^	*U*^33^	*U*^12^	*U*^13^	*U*^23^
O2	0.0660 (7)	0.0800 (9)	0.0650 (8)	−0.0019 (6)	0.0283 (6)	0.0010 (6)
O4	0.0655 (7)	0.0787 (9)	0.0648 (8)	0.0073 (6)	0.0321 (6)	−0.0021 (6)
O1	0.0620 (7)	0.0813 (9)	0.0663 (8)	−0.0011 (6)	0.0244 (6)	−0.0140 (6)
C7	0.0654 (9)	0.0671 (11)	0.0449 (8)	0.0118 (8)	0.0173 (7)	0.0042 (7)
O3	0.0943 (10)	0.1107 (13)	0.0621 (8)	−0.0072 (8)	0.0427 (7)	−0.0087 (7)
C5	0.0562 (8)	0.0627 (10)	0.0464 (8)	0.0083 (7)	0.0192 (6)	0.0048 (7)
C4	0.0580 (9)	0.0631 (10)	0.0500 (8)	0.0089 (7)	0.0169 (7)	0.0027 (7)
C8	0.0615 (9)	0.0602 (10)	0.0516 (9)	0.0086 (7)	0.0162 (7)	0.0035 (7)
C6	0.0659 (10)	0.0709 (12)	0.0552 (10)	0.0076 (8)	0.0244 (8)	0.0058 (8)
C3	0.0568 (9)	0.0811 (13)	0.0635 (10)	0.0024 (8)	0.0219 (8)	−0.0051 (9)
C13	0.0696 (11)	0.0759 (13)	0.0609 (10)	0.0036 (9)	0.0157 (8)	−0.0028 (9)
C9	0.0817 (12)	0.0749 (13)	0.0558 (10)	−0.0003 (10)	0.0199 (9)	0.0067 (8)
F1	0.0936 (9)	0.1271 (13)	0.1624 (16)	−0.0198 (9)	0.0608 (10)	0.0147 (10)
C10	0.0994 (15)	0.0889 (16)	0.0738 (13)	0.0043 (12)	0.0424 (12)	0.0138 (11)
C2	0.0683 (11)	0.0995 (16)	0.0783 (13)	0.0120 (11)	0.0121 (10)	0.0050 (12)
C12	0.0655 (11)	0.0870 (15)	0.0845 (14)	−0.0003 (10)	0.0130 (10)	−0.0014 (11)
C11	0.0621 (10)	0.0815 (14)	0.1091 (16)	0.0033 (10)	0.0377 (11)	0.0108 (13)
C1	0.0819 (13)	0.0876 (16)	0.1103 (18)	−0.0143 (11)	0.0337 (12)	−0.0196 (13)

### Geometric parameters (Å, °)

**Table:**

O2—C6	1.348 (2)	C13—C12	1.379 (3)
O2—C3	1.432 (2)	C13—H13A	0.9300
O4—C4	1.2063 (19)	C9—C10	1.370 (3)
O1—C4	1.347 (2)	C9—H9A	0.9300
O1—C3	1.4388 (19)	F1—C11	1.367 (2)
C7—C5	1.349 (2)	C10—C11	1.362 (3)
C7—C8	1.451 (2)	C10—H10A	0.9300
C7—H7A	0.9300	C2—H2A	0.9600
O3—C6	1.202 (2)	C2—H2B	0.9600
C5—C4	1.465 (2)	C2—H2C	0.9600
C5—C6	1.488 (2)	C12—C11	1.356 (3)
C8—C9	1.391 (2)	C12—H12A	0.9300
C8—C13	1.392 (3)	C1—H1A	0.9600
C3—C1	1.491 (3)	C1—H1B	0.9600
C3—C2	1.516 (3)	C1—H1C	0.9600
			
C6—O2—C3	118.82 (14)	C8—C13—H13A	119.1
C4—O1—C3	120.31 (14)	C10—C9—C8	121.03 (19)
C5—C7—C8	133.77 (16)	C10—C9—H9A	119.5
C5—C7—H7A	113.1	C8—C9—H9A	119.5
C8—C7—H7A	113.1	C11—C10—C9	118.7 (2)
C7—C5—C4	126.02 (15)	C11—C10—H10A	120.6
C7—C5—C6	115.63 (15)	C9—C10—H10A	120.6
C4—C5—C6	117.82 (15)	C3—C2—H2A	109.5
O4—C4—O1	116.92 (15)	C3—C2—H2B	109.5
O4—C4—C5	126.49 (16)	H2A—C2—H2B	109.5
O1—C4—C5	116.36 (14)	C3—C2—H2C	109.5
C9—C8—C13	117.60 (18)	H2A—C2—H2C	109.5
C9—C8—C7	125.56 (16)	H2B—C2—H2C	109.5
C13—C8—C7	116.70 (15)	C11—C12—C13	117.8 (2)
O3—C6—O2	118.56 (17)	C11—C12—H12A	121.1
O3—C6—C5	124.86 (18)	C13—C12—H12A	121.1
O2—C6—C5	116.53 (15)	C12—C11—C10	123.1 (2)
O2—C3—O1	109.70 (13)	C12—C11—F1	118.2 (2)
O2—C3—C1	106.49 (17)	C10—C11—F1	118.6 (2)
O1—C3—C1	106.23 (16)	C3—C1—H1A	109.5
O2—C3—C2	110.14 (16)	C3—C1—H1B	109.5
O1—C3—C2	109.73 (16)	H1A—C1—H1B	109.5
C1—C3—C2	114.39 (16)	C3—C1—H1C	109.5
C12—C13—C8	121.71 (19)	H1A—C1—H1C	109.5
C12—C13—H13A	119.1	H1B—C1—H1C	109.5

### Hydrogen-bond geometry (Å, °)

**Table:**

*D*—H···*A*	*D*—H	H···*A*	*D*···*A*	*D*—H···*A*
C10—H10A···O1^i^	0.93	2.47	3.373 (3)	164

Symmetry codes: (i) −*x*+1, *y*+1/2, −*z*−1/2.
